# Disrupted topological organization of structural and functional brain connectomes in clinically isolated syndrome and multiple sclerosis

**DOI:** 10.1038/srep29383

**Published:** 2016-07-12

**Authors:** Ni Shu, Yunyun Duan, Mingrui Xia, Menno M. Schoonheim, Jing Huang, Zhuoqiong Ren, Zheng Sun, Jing Ye, Huiqing Dong, Fu-Dong Shi, Frederik Barkhof, Kuncheng Li, Yaou Liu

**Affiliations:** 1 State Key Laboratory of Cognitive Neuroscience and Learning and IDG/McGovern Institute for Brain Research, Beijing Normal University, Beijing 100875, P. R. China; 2 Center for Collaboration and Innovation in Brain and Learning Sciences, Beijing Normal University, Beijing 100875, P. R. China; 3 Department of Radiology, Xuanwu Hospital, Capital Medical University, Beijing 100053, P. R. China; 4 Department of Radiology and Nuclear Medicine, Neuroscience Campus Amsterdam, VU University Medical Center, Amsterdam 1007 MB, The Netherlands; 5 Department of Anatomy and Neuroscience, VU University Medical Center, Amsterdam 1007 MB, The Netherlands; 6 Department of Neurology, Xuanwu Hospital, Capital Medical University, Beijing 100053, P. R. China; 7 Department of Neurology and Tianjin Neurological Institute, Tianjin Medical University General Hospital, Tianjin 300052, P. R. China

## Abstract

The brain connectome of multiple sclerosis (MS) has been investigated by several previous studies; however, it is still unknown how the network changes in clinically isolated syndrome (CIS), the earliest stage of MS, and how network alterations on a functional level relate to the structural level in MS disease. Here, we investigated the topological alterations of both the structural and functional connectomes in 41 CIS and 32 MS patients, compared to 35 healthy controls, by combining diffusion tensor imaging and resting-state functional MRI with graph analysis approaches. We found that the structural connectome showed a deviation from the optimal pattern as early as the CIS stage, while the functional connectome only showed local changes in MS patients, not in CIS. When comparing two patient groups, the changes appear more severe in MS. Importantly, the disruptions of structural and functional connectomes in patients occurred in the same direction and locally correlated in sensorimotor component. Finally, the extent of structural network changes was correlated with several clinical variables in MS patients. Together, the results suggested early disruption of the structural brain connectome in CIS patients and provided a new perspective for investigating the relationship of the structural and functional alterations in MS.

Multiple sclerosis (MS) is an inflammatory demyelinating disease of the central nervous system (CNS)^1^. The first manifestation of most MS is an acute or subacute episode of optic neuritis (ON), a brain-stem/cerebellar syndrome or a spinal cord syndrome. This initial episode is known as a clinically isolated syndrome (CIS)^2^. The early identification of the brain alterations in CIS is important for understanding the pathophysiology of the disease and may provide possible guidance for early treatment to prevent axon pathology and slow the disease progression^2^.

A multitude of neuroimaging studies have reported disease effects in the brain in MS, including CIS, such as brain atrophy^3^,^4^,^5^, diffusion abnormalities^6^,^7^,^8^,^9^,^10^ or functional damage and plasticity^11^,^12^,^13^,^14^,^15^. However, the brain is an integrative complex network, and it is claimed that clinical disability and cognitive impairment in neurological diseases can be understood from the perspective of the brain’s topology^16^,^17^. Therefore, analyzing network topology in MS, especially the earliest stage (CIS), is essential for understanding the underlying disease mechanism and identifying important biomarkers for clinical disability or cognitive impairment.

Graph theory provides a comprehensive and sophisticated framework to characterize network topology^18^. Structural and functional network studies have been applied in several previous MS studies^19^,^20^,^21^,^22^,^23^,^24^,^25^,^26^,^27^. A structural network based on T1-weighted MRI images identified disrupted integration in MS, proportional to the white matter (WM) lesion load^23^, while diffusion tensor imaging (DTI) network analysis showed decreased global and local efficiency in MS correlated with clinical disability^21^. Furthermore, functional networks based on magnetoencephalography (MEG) display a more regular network in MS patients^24^,^28^. More specifically, temporal regions appear to lose “hubness”, while parietal regions appear to become more hub-like in early MS patients^20^. Additionally, network analysis based on resting-state functional MRI (rs-fMRI) found abnormal network properties in MS patients, which can contribute to distinguishing cognitively impaired MS patients from healthy controls^22^.

Despite of the advances, it remains unknown how the network changes in CIS and how network alterations on a functional level relate to network changes on a structural level, highlighting the need for combined structural and functional network studies in CIS and MS. Thus, the aim of this study was to provide a comprehensive view of the whole-brain structural and functional connectome and demonstrate its relationship based on DTI and rs-fMRI in CIS and MS combined with graph theory analyses. We set out to assess (i) whether structural and functional networks are affected in the earliest stage of MS (CIS), (ii) the changes of structural and functional networks from CIS to MS, and (iii) the relationship between structural and functional networks and its association with clinical disability and cognitive impairment.

## Methods

### Participants

This study included 41 CIS patients (26 females; mean age 35.7 ± 10.7 years) (optic neuritis, n = 18; spinal cord syndrome, n = 16; brainstem syndrome n = 5; cerebellar syndrome, n = 2), 32 relapsing-remitting MS (RRMS) patients (24 females; mean age 34.8 ± 8.3 years) and 35 healthy controls (23 females; mean age 35.0 ± 11.5 years). All CIS patients were prospectively examined within 6 months from onset according to the following criteria: (1) a single clinical episode suggestive of MS^29^; (2) exclusion of other possible diagnoses; (3) sufficient image quality. All MS patients were RRMS patients diagnosed according to the 2010 modified McDonald Criteria^30^. In addition, the healthy controls were included without history of neurological dysfunction and with normal findings on neurological examination. All of the subjects were right-handed, as measured by the Edinburgh Inventory^31^. The main demographic and clinical characteristics, including the Expanded Disability Status Scale (EDSS) score^32^ and disease duration of the patients, were assessed by an experienced neurologist (J.Y.). Additionally, the Mini-Mental State Examination (MMSE) and Paced Auditory Serial Attention Test (PASAT2 and PASAT3 versions), exploring attention and information processing, were also assessed. The main demographic and clinical information of all the participants is presented in Table 1.

This study was approved by the Institutional Review Board of Xuanwu Hospital, Capital Medical University. Written informed consent was obtained from each participant. All the methods were carried out in accordance with the approved guidelines.

### MRI data acquisition

The MRI data were acquired using a SIEMENS TRIO 3.0 T scanner in the Department of Radiology, Xuanwu Hospital. All participants underwent high-quality MRI scanning, which included a 3D T1-weighted magnetization prepared rapid acquisition gradient echo (MPRAGE) scan [176 sagittal slices, slice thickness = 1 mm, repetition time (TR) = 1600 ms, echo time (TE) = 2.13 ms, field of view (FOV) = 224 × 256 mm^2^, voxel size of 1 mm^3^ isotropic]; a T2-weighted MRI scan (35 axial slices, slice thickness = 4 mm, TR = 5000 ms, TE = 87 ms, FOV = 256 × 256 mm^2^, voxel size = 1 × 1 × 4 mm^3^); a DTI scan (60 axial slices, slice thickness = 2 mm, 30 diffusion directions with b = 1000 s/mm^2^, and an additional b0 image, TR = 11000 ms, TE = 98 ms, FOV = 256 × 256 mm^2^, voxel size of 2 mm^3^ isotropic, average = 2); and a rs-fMRI scan (32 axial slices, slice thickness = 3 mm, slice gap = 1 mm, TR = 2000 ms, TE = 30 ms, flip angle = 90°, FOV = 220 × 220 mm^2^, voxel size = 3.4 × 3.4 × 3 mm^3^, 180 image volumes).

### MRI data preprocessing

#### DTI data preprocessing

The preprocessing procedure for the DTI data included eddy current and motion artifact correction, estimation of the diffusion tensor and calculation of the fractional anisotropy (FA). First, eddy current distortions and the motion artifacts in the DTI dataset were corrected by applying an affine alignment of the diffusion-weighted images to the b0 image. The b-matrix was accordingly reoriented based on the transformation matrix. After this process, the diffusion tensor was estimated by solving the Stejskal and Tanner equation^33^, and the reconstructed tensor matrix was diagonalized to obtain 3 eigenvalues (λ1, λ2, λ3) and their corresponding eigenvectors. The FA value of each voxel was also calculated^34^. The preprocessing of the DTI data was performed using the FMRIB Diffusion Toolbox (FDT) in FSL (fsl.fmrib.ox.ac.uk/fsl/fslwiki/FDT).

#### rs-fMRI data preprocessing

The preprocessing of the rs-fMRI data included motion correction, brain extraction, spatial smoothing, band-pass filtering (0.01–0.1 Hz) the data and regressing out nuisance covariates, including six rigid body motion parameters, volumes corresponding to motion spikes, and average WM, cerebrospinal fluid (CSF), and global time series. The first 10 functional volumes were discarded to allow stabilization of the initial signal and adaptation of the participants to the circumstances. The preprocessing of the rs-fMRI data was performed using the SPM8 (www.fil.ion.ucl.ac.uk/spm) and DPARSF software (www.restfmri.net)^35^.

#### Measurement of WM lesion load

Hyperintense WM lesions of each patient were manually delineated on the T2-weighted images by an experienced radiologist (Y.L.) who was blind to the clinical details. Then, the total WM lesion load (TWMLL) for each patient was calculated. The lesions were re-delineated on two separate occasions (at least 3-months apart) in ten of the patients, and the intra-rater reliability was 95.2%.

### Network construction

Nodes and edges are the two fundamental elements of a network. In this study, we constructed individual structural and functional connectomes using the following procedures.

#### Network node definition

**Figure 1:**
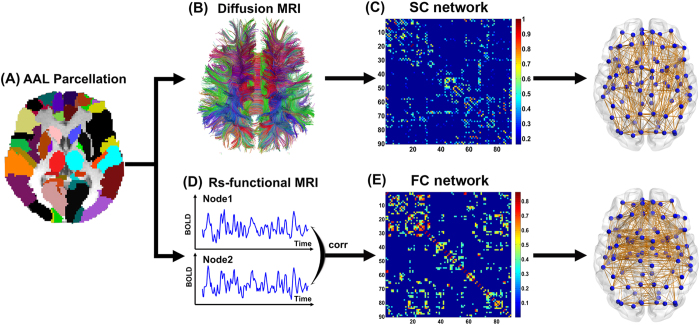
The flowchart of structural and functional connectome construction. (**A**) Individual T1 images and AAL template were used for automatic parcellation of the cortex into 90 brain regions, forming the nodes of the individual brain networks. (**B**) Streamline tractography was applied to the diffusion MRI data to reconstruct the white matter pathways. From the set of reconstructed streamlines, the streamlines that interconnected regions *i* and *j* from the set of 90 regions were taken as an edge between nodes *i* and *j* in the structural brain network. The streamline count was taken to represent the weight of the connection and was aggregated into a structural connectivity (SC) matrix (**C**). (**D**) Functional connectivity (FC) between nodes *i* and *j* was computed as the level of correlation between their resting-state functional magnetic resonance imaging (fMRI) and blood oxygenation level dependent (BOLD) time series, resulting in a matrix, FC, with only significantly positive correlation values (**E**). The matrices and 3D representations (lateral view) of the SC and FC networks of a representative healthy subject were shown in the right panel. The nodes are located according to their centroid stereotaxic coordinates, and the edges are coded according to their connection weights. For details, see the Materials and Methods section.

The Automated Anatomical Labeling (AAL) template^36^ was used to define the network nodes. Briefly, the individual T1-weighted images were coregistered to the b0 images in the DTI space. The transformed T1 images were then nonlinearly transformed to the ICBM152 T1 template in the Montreal Neurological Institute (MNI) space. Inverse transformations were used to warp the AAL atlas from the MNI space to the DTI native space. Using this procedure, we obtained 90 cortical and subcortical ROIs (45 for each hemisphere, see Table S1), each representing a node of the network (Fig. 1). To ensure the consistency of the brain parcellation maps, all rs-fMRI images were also coregistered with the b0 images. The whole procedure was performed using the SPM8 software.

#### Structural connectome

First, diffusion MRI tractography was performed using Diffusion Toolkit (www.trackvis.org/dtk) based on the “fiber assignment by continuous tracking” method^37^. All tracts in the DTI dataset were computed by seeding each voxel with an FA greater than 0.2. The tractography was terminated if it turned an angle greater than 45 degrees or reached a voxel with an FA less than 0.2^37^. As a result, all the fiber pathways in the brain were constructed using the deterministic tractography method. To define the network edges, we selected a threshold of 3 fiber streamlines^21^. For each pair of ROIs i and j, we defined the fiber number (FN) of the connected fibers with two end points located in these two regions as the weight of the edge between ROIs i and j. Therefore, for each participant, an FN-weighted 90 × 90 structural connectivity (SC) network was constructed (Fig. 1).

#### Functional connectome

Based on the brain parcellation map and coregistered rs-fMRI images, we calculated the mean rs-fMRI time series by averaging over the time-series of all voxels contained within each ROI. To measure the interregional resting-state functional connectivity (RSFC), we calculated the Pearson correlation coefficient of the mean time-series between any pair of ROIs and estimated their corresponding significance levels (i.e., p values). Negative correlation coefficients were set to zero^38^, and a threshold was applied to the connection significance to remove spurious functional connectivity (p < 0.05, Bonferroni correction). All the above procedures were performed using the DPARSF software^35^. Then, a weighted 90 × 90 functional connectivity (FC) network was constructed for each participant (Fig. 1).

### Network analysis

Different levels of network analyses were performed, including global network metrics, regional nodal properties and local connections. For the global network metrics, we quantified the network strength (S_p_), global efficiency (E_glob_), local efficiency (E_loc_), shortest path length (L_p_), clustering coefficient (C_p_) and small-world parameters (λ, γ and σ)^39^. For the regional characteristics, we considered the nodal efficiency^40^. All network analyses were performed using the GRETNA software (www.nitrc.org/projects/gretna) and visualized using the BrainNet Viewer software (www.nitrc.org/projects/bnv). Detailed definitions of the network metrics are provided in Supplement 1.

### Statistical analysis

#### Between group differences

Demographic factor and cognitive scores including age, MMSE, PASAT2 and PASAT3 among three groups were compared using one-way analysis of variance (ANOVA). Gender distribution was compared with the χ^2^ test. The clinical variables including disease duration and TWMLL between the CIS and MS groups were compared using two-sample t-test and the EDSS scores were compared with Wilcoxon rank sum test. Group comparisons of global and local network metrics were performed with one-way analysis of covariance (ANCOVA) with age and gender as covariates, and *post-hoc* pairwise comparisons were performed by a general linear regression model if ANCOVA yielded significant results (p < 0.05).

#### Network-based statistic (NBS)

To localize the specific connected components in which the structural or functional connections differed between each pair of groups, we used a NBS approach^41^ for both the SC and FC networks. We first detected the significant nonzero connections (backbone) within each group by performing a nonparametric one-tailed sign test (p < 0.05, corrected). Next, the nonzero connections within either the patient or control groups were detected and combined into a connection mask. The NBS approach was then conducted within the connection mask, where a primary threshold (p = 0.05) was first applied to a t statistic (two-sample one-tailed t tests). This t statistic was computed for each link to define a set of suprathreshold links among which any connected components and their size (number of links) could then be determined. To estimate the significance for each component, the null distribution of the connected component size was empirically derived using a nonparametric permutation approach (10,000 permutations). For each permutation, all of the subjects were randomly reallocated into two groups, and the t statistic was computed independently for each link. Next, the threshold (p = 0.05) was used to generate suprathreshold links among which the maximal connected component size was recorded. Finally, for a connected component of size M that was found in the right grouping of controls and patients, the corrected p value was determined by calculating the proportion of the 10,000 permutations for which the maximal connected component was larger than M. Of note, the effects of age and gender were removed by a regression analysis performed before the statistical analysis of connections. For a detailed description, see Zalesky *et al*.^41^.

#### Relationship between structural and functional alterations

For the overlapping structural and functional alterations, we performed a partial correlation analysis between the SC and FC values across all patients, removing the effects of age and gender.

#### Relationship between network metrics and clinical variables

Multiple linear regressions were used to assess the relationships between altered network metrics and clinical variables (disease duration, TWMLL, EDSS, MMSE, PASAT2 and PASAT3) in the CIS and MS groups separately, removing the effects of age and gender.

## Results

### Demographic and clinical characteristics

There were no significant differences in age or gender distribution among three groups. As for the clinical variables, both the CIS and MS patient groups exhibited lower MMSE, PASAT2 and PASAT3 scores than the controls, and the MS patients exhibited lower MMSE and PASAT scores than the CIS group (all p < 0.05). As expected, the MS patients had larger TWMLL, longer disease duration and higher EDSS scores than the CIS patients (all p < 0.05) (Table 1).

### Group differences in global network metrics

**Figure 2:**
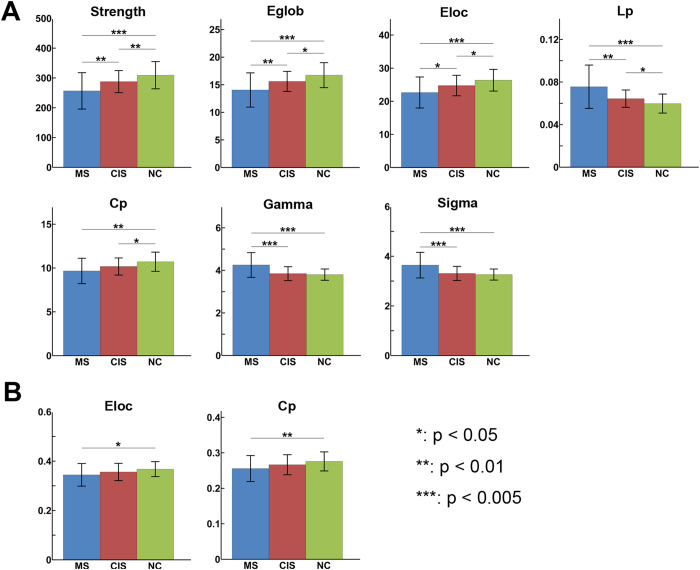
Group differences in the global network metrics of structural and functional connectomes. The bar and error bar represent the mean values and standard deviations of the network properties in each group after removing the effects of age and gender. (**A**) Significantly reduced strength, global efficiency, local efficiency, clustering and increased shortest path length of SC networks were observed in both CIS and MS patients relative to the controls. (**B**) Reduced local efficiency and clustering of FC networks in MS patients relative to controls were found. *p < 0.05; **p < 0.01; ***p < 0.005.

Both the structural and functional brain networks of the controls and patients showed prominent small-world properties (lambda ≈ 1, gamma > 1), illustrating the balance of information integration and segregation of the human brain. For the SC network, ANCOVA revealed significant group differences in most global network metrics, including network strength (p = 10^−4^), global efficiency (p < 10^−4^), local efficiency (p = 0.0004), shortest path length (p < 10^−4^), clustering coefficient (p = 0.002), gamma (p < 10^−4^) and sigma (p < 10^−4^) (Fig. 2A). *Post-hoc* analyses revealed that both CIS and MS patients exhibited decreased network strength, global and local efficiency and clustering, and increased shortest path length compared with the controls (all p < 0.05). Moreover, the MS patients showed increased gamma (p = 0.0001) and sigma (p = 0.0002) compared to the controls. Compared to the CIS patients, the MS patients showed reduced network strength, global and local efficiency and increased shortest path length, gamma and sigma (all p < 0.05).

For the FC network, significant group differences were found only in the local efficiency (p = 0.045) and clustering coefficient (p = 0.032) (Fig. 2B). *Post-hoc* analyses revealed that the MS patients showed decreased local efficiency (p = 0.019) and clustering (p = 0.015) compared to the controls. No significant differences in any global metrics were found between the CIS group and the other two groups (all p > 0.05).

### Group differences in nodal efficiency

**Figure 3:**
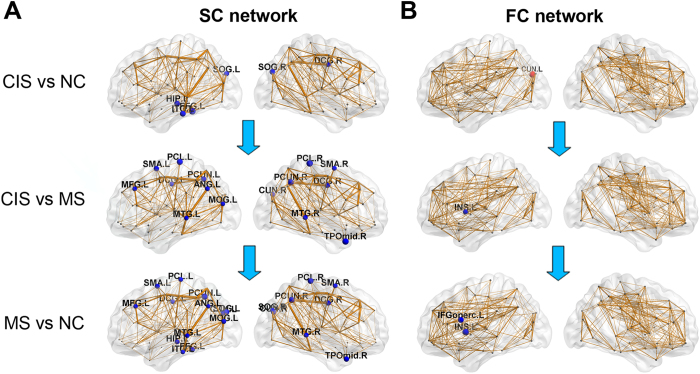
Distributed brain regions with significant differences among three groups. The node sizes indicate the significance of between-group differences in the regional efficiency. (**A**) For the SC network, nodes in blue showed reduced efficiency in CIS and MS patients compared with controls, and decreased efficiency in MS compared with CIS (*p* < 0.05, corrected). (**B**) For the FC network, nodes in red showed increased efficiency in CIS compared with controls and nodes in blue showed decreased efficiency in MS compared with CIS or controls (*p* < 0.01, uncorrected). The network shown here was constructed by averaging the connection matrices across all subjects and thresholded with a sparsity of 10%. The nodal regions are located according to their centroid stereotaxic coordinates. The surface visualization of the WM networks was accomplished using the BrainNet Viewer software (www.nitrc.org/projects/bnv).

For the SC network, 21 regions showed significant group differences in nodal efficiency (p < 0.05, corrected), widely distributed in the frontal, parietal, temporal, and occipital cortices (Fig. 3A). The MS patients exhibited reduced nodal efficiency in all of those regions compared to the controls (p < 0.05, corrected). The CIS patients showed decreased nodal efficiency, restricted to the temporal and occipital regions (p < 0.05, corrected). Between the CIS and MS patients, regions with decreased efficiency in MS were mainly located in the parietal and temporal cortices, symmetrically distributed along the cortical midline, including the bilateral precuneus, bilateral paracentral lobule, bilateral supplemental motor area, bilateral middle temporal gyrus, and bilateral middle cingulate gyrus (p < 0.05, corrected).

For the FC network, no regional differences survived multiple comparison correction. A trend of group differences was found in the left insular, left cuneus and left opercular parts of the inferior frontal gyrus (IFGoper) (p < 0.01, uncorrected) (Fig. 3B). Compared with controls, MS patients showed decreased efficiency in the left insular and left IFGoper, while the CIS patients showed increased efficiency in the left cuneus and decreased efficiency in the left IFGoper (all p < 0.05). Relative to the CIS patients, MS patients showed decreased efficiency in the left insular and left cuneus (all p < 0.05).

### Group differences in structural and functional connectivity

**Figure 4:**
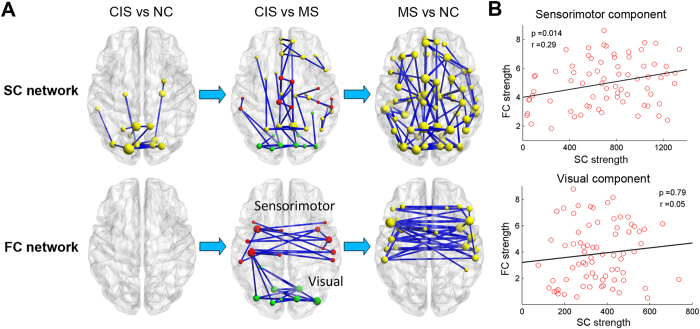
Disrupted structural and functional components in CIS and MS patients. (**A**) The top panel represents connected networks showing decreased structural connections in CIS vs. controls, MS vs. CIS and MS vs. controls. The regional pairs showed decreased connections in the patient groups (*p* < 0.05, corrected). The bottom panel represents the connected networks, showing decreased functional connections in MS vs. CIS and MS vs. controls (*p* < 0.05, corrected). Between CIS and controls, no connected components with significant differences were found. The nodes in red and green represent regions within sensorimotor and visual systems respectively, according to He *et al*.^23^. The nodes and connections were mapped onto the cortical surfaces using the in-house BrainNet viewer software. (**B**) Correlation between the structural and functional connection strength within sensorimotor and visual components across all patients while removing the effects of age and gender.

We used NBS analysis to identify disrupted connected components in patients. For the SC network, a single connected network with 15 nodes and 14 connections was altered in CIS patients compared to the controls (p = 0.048, corrected), comprising the bilateral occipital regions, parahippocampus and precuneus (Fig. 4A). For the MS patients, a single connected network consisting of 67 nodes and 98 edges was altered (p < 10^−4^, corrected), which were widely distributed across the whole brain (Fig. 4A). Importantly, all of the connections exhibited decreased values in the patients compared with the controls. Relative to the CIS group, the MS patients showed a disrupted network composed of 47 nodes and 55 edges (p = 0.0008, corrected), mainly involved in bilateral homotopic regions along the cortical midline (Fig. 4A).

For the FC network, a disrupted network with 22 nodes and 38 connections was identified in MS patients (p = 0.0066, corrected), involving the bilateral lateral frontal and sensorimotor areas (Fig. 4A). No disrupted FC components were found in CIS. Between the CIS and MS patients, MS showed a decreased network with 22 nodes and 30 connections (p = 0.024, corrected), mainly composed of bilateral visual and sensorimotor areas (Fig. 4A).

### Relationship between structural and functional alterations

From the NBS analysis, we found that overlapping alterations between the disrupted SC and FC mainly involved in the sensorimotor and visual areas in the patient group. According to He *et al*.^42^, the disrupted FC components between CIS and MS were divided into two functional modules/systems (sensorimotor and visual components). A significant correlation was found between SC and FC reduction within the sensorimotor component (r = 0.28; p = 0.014), but not for the visual component (p > 0.1) (Fig. 4B).

### Relationship between network metrics and clinical variables

**Figure 5:**
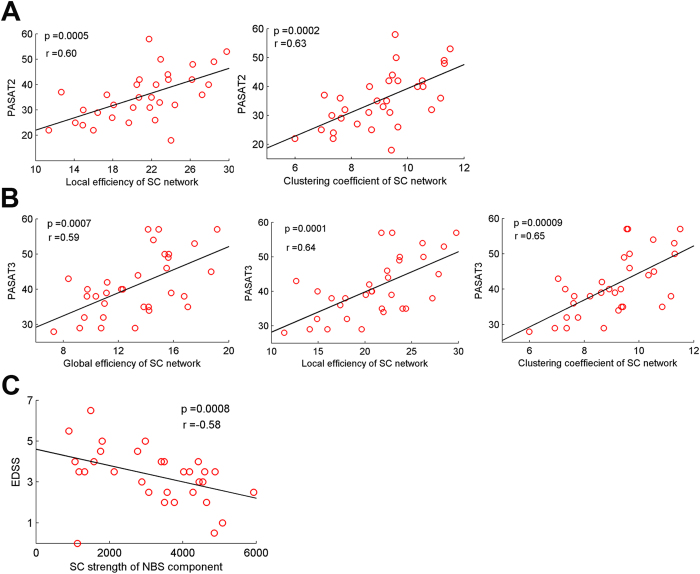
Correlations between network metrics and clinical variables in patients with MS. Plots showing the linear correlation between altered network metrics with PASAT2 (**A**), PASAT3 (**B**), and EDSS (**C**) scores in MS patients (all p < 0.05, corrected). The red dots represent the adjusted values of MS patients after controlling for age and gender.

For the clinical relevance, significant clinical correlations were found only in the MS group but not in the CIS group. First, the local efficiency and clustering coefficient of SC network were correlated with the PASAT2 in MS (all p < 0.05, corrected) (Fig. 5A). Second, the global efficiency, local efficiency and clustering coefficient of the SC network were correlated with the PASAT3 score (all p < 0.05, corrected) (Fig. 5B). And decreased strength of the NBS component of SC network was correlated with increased EDSS score in MS patients (p < 0.05, corrected) (Fig. 5C). Additionally, the TWMLL was also correlated with several network metrics of SC network, including network strength, global efficiency, and shortest path length in MS patients (all p < 0.05, uncorrected), which are consistent with the findings of our previous study^21^. No significant correlations were observed between network metrics and clinical variables in the CIS group.

## Discussion

In this study, we investigated the topological alterations of both the structural and the functional connectomes in CIS and MS patients by combined use of DTI and rs-fMRI with graph theoretical analysis. Our main findings can be summarized as follows. First, the structural connectome showed deviation from the optimal pattern in MS, even in the early phase of the disease (CIS), while the functional connectome only showed local changes in the MS stage. Second, network changes appear more severe in MS compared to CIS. Third, the structural and functional disruptions were in the same direction and locally correlated, suggesting that they are partly independent and provide complementary information by different modalities. Finally, the extent of network changes was correlated with cognitive impairment and physical disability in MS patients but not in CIS patients.

### Disrupted topological efficiency of structural connectome in CIS and MS

The human brain is a complex system with an optimal balance between local specialization and global integration. We identified the small-world properties of both structural and functional networks in CIS patients, MS patients and controls, which were characterized by both high global and local efficiencies^18^. Although the small-world properties were preserved in the MS connectome, both the global and local efficiencies in the structural networks were significantly decreased compared with the controls. The decreases in both global and local efficiencies reflect the disrupted topological organization of the WM networks in patients with MS, which could be due to diffusively impaired structural connections, including both long- and short-distance connections. Previous results from graph analysis based on structural and diffusion MRI revealed similar topological alterations of the structural networks in MS^21^,^23^,^25^. We extend our findings to CIS, the earliest stage of MS. In CIS patients, decreased global and local efficiency was already emerging, and all of the network properties of CIS patients exhibited intermediate values between the controls and MS, suggesting a transition stage of the disease.

In CIS, the most affected brain regions and structural connections were located within the visual areas (e.g., the superior occipital gyrus) and default-mode network (DMN) (e.g., the precuneus). As visual deficits are among the main symptoms for CIS patients in our cohort, the brain structural changes in occipital lobe were consistent with the clinical deficits through transsynaptic degeneration. For the DMN alterations, previous DTI studies have demonstrated similar patterns of alteration in both CIS and MS patients^12^,^43^, suggesting DMN disruption as an early biomarker of brain damage in MS.

Between the CIS and MS patients, we found the most significant differences in structural networks largely overlapped with the important core regions (hubs) and their connections of the structural connectome, such as the bilateral precuneus, anterior and middle cingulate gyrus^44^. With disease progression, hub regions are the most vulnerable ones due to their wide connections to other regions. The important roles of hub regions in information integration make them “symptomatic”^44^. Recently, the “hub overload and failure” theory was proposed as a common pathway of different neurological disorders^27^,^45^. Our results suggested that hubs were not the earliest to be affected during the onset phase of MS but were the most severely disrupted with disease progression and may be crucial for understanding the mechanisms of disease conversion and clinical disability.

### Topological alterations of functional connectome in CIS and MS

In contrast to the consistent findings in structural network studies, previous graph analyses of the functional connectome have shown discordant results. Both decreased network efficiency and no changes in functional networks have been reported in MS^22^,^24^,^46^,^47^,^48^,^49^. In this study, only MS showed significantly decreased local efficiency compared to the controls, no significant changes in any global network metrics were identified in CIS, suggesting that functional changes were subtle during the early stage of the disease. The subtle functional network changes may be due to the coexistence of both increased (functional plasticity) and decreased (functional disconnection) connections^49^,^50^.

In the functional connectivity analysis, several components with decreased functional connectivity were identified in MS but not in CIS. The main differences between MS and CIS were located in the homotopic FC within the sensorimotor and visual networks, consistent with the previous findings of inter-hemispheric FC alteration in MS^51^,^52^, possibly due to corpus callosum damage and indirect structural connectivity of homotopic regions.

### Relationship between altered structural and functional connectomes

We determined that the topological changes for both the structural and functional networks in CIS and MS progressed in the same direction. CIS showed an intermediate transition phase between MS and the controls for both modalities. Certain local connections, such as the sensorimotor and visual areas, were reduced in both types of networks. Positive correlations between SC and FC were found for certain short-distance connections within the sensorimotor network, suggesting the possible structural substrate of functional deficits between parts of the SC and FC, but these results need to be further validated.

However, in contrast to the widespread structural changes, mild functional alterations were found in the patients, especially during the early stage (CIS) of the disease, indicating that the functional changes cannot be simply explained by the structural changes. As suggested by previous studies, the relationship between SC and FC in the healthy human brain is complicated, not a simply one-to-one correspondence^53^,^54^,^55^,^56^,^57^. In disease states, the relationship between brain structures and functions may be more complicated and require modeling studies for further exploration.

Certain factors may contribute to this discrepancy. Alterations in the functional network may be secondary to the structural network changes, which might need to achieve a threshold. Another possible explanation is that functional changes may be less stable or robust than structural changes in MS, as we know that brain functions should be studied from a dynamic perspective^58^, or that the functional changes in MS are more complex, with dynamic changes, and presumably might be a mixed outcome of structural alterations with other factors such as brain plasticity^11^. To better understand the associations between structural damage and functional impairment and their interplay during the different stages of the disease, a theoretical framework using various computational and modeling methods^59^,^60^ should be applied to longitudinal datasets.

### Clinical relevance of network alterations in CIS and MS

Cognitive scores (PASAT2 and PASAT3) were correlated with SC network metrics, suggesting that the SC network may provide potential biomarkers for assessing and monitoring cognitive impairment in MS, although the underlying neural substrate needs further elucidation. SC network metrics (network strength of the NBS component) also showed moderate correlation with EDSS, indicating that SC networks could also be assessed for evaluating physical disability in MS. A strong correlation trend was observed among several SC network metrics, such as global efficiency and local efficiency and TWMLL, suggesting that lesions are a key factor contributing to network abnormalities in CIS and MS. No significant correlations between network metrics and clinical variables were observed in CIS, implying that the network changes in CIS may represent a transitional phase.

### Methodological issues

Several limitations should be addressed. First, the samples were obtained from a cross-sectional design, whereas future studies with longitudinal MRI data will be required to validate the cross-sectional findings of disease progression. Second, deterministic tractography was used for the reconstruction of WM tracts, which may result in the loss of existing fibers due to the WM lesions in the patients or “fiber crossing” problem^61^. Future studies should employ more advanced tractography techniques, such as probabilistic tractography, to define the network edges^62^. Third, comprehensive neuropsychological tests for MS patients should be examined to evaluate the relationship between brain network alterations and cognitive impairment in different cognitive domains. Finally, CIS is a heterogeneous group, which is reflected by the patients with different syndromes (optic neuritis, spinal cord syndrome, brainstem syndrome and cerebellar syndrome). The combination of the heterogeneous patients into one group may consequently negatively affect the statistical findings. Further study is warranted to investigate the specific structural and functional network changes in homogenous CIS group with large sample size (e.g. optic neuritis or spinal cord syndrome).

## Conclusion

Our study demonstrated disrupted structural connectome in the earliest stage of MS, while functional networks remain stable at that stage. Both the structural and functional disruptions occurred in the same direction in MS and they were locally correlated, suggesting that partly independent and complementary information can be provided by different modalities. Between CIS and MS, disrupted hub regions and connections were identified, highlighting the key role of the disrupted core component of brain connectome as a possible pathological substrate of disease conversion. Importantly, certain structural network metrics correlated with clinical variables, suggesting potential connectome-based biomarkers for predicting cognitive impairment and physical disability in MS patients.

## Additional Information

**How to cite this article**: Shu, N. *et al*. Disrupted topological organization of structural and functional brain connectomes in clinically isolated syndrome and multiple sclerosis. *Sci. Rep.* **6**, 29383; doi: 10.1038/srep29383 (2016).

## Supplementary Material

Supplementary Information

## Figures and Tables

**Table 1:** The demographic information and clinical characteristics of all participants.

	Controls (n = 35)	CIS (n = 41)	MS (n = 32)	F/T/χ2 /Z value	P value
Mean Age (years)	35.0 ± 11.5	35.7 ± 10.7	34.8 ± 8.3	0.08#	0.92^#^
Gender (F/M)	23/12	26/15	24/8	1.17^╫^	0.56^╫^
Mean MMSE	29.1 ± 1.3	27.6 ± 1.4	25.9 ± 1.8	36.44^#^	<0.001^#^
Mean PASAT2	46.7 ± 9.2	39.4 ± 7.7	35.4 ± 9.8	14.14^#^	<0.001^#^
Mean PASAT3	53.9 ± 6.2	47.5 ± 7.6	41.0 ± 8.7	24.38^#^	<0.001^#^
Mean disease duration (months)	–	2.6 ± 2.5	41.8 ± 28.7	8.73^*^	<0.001^*^
Mean TWMLL (ml)	–	4.5 ± 10.6	10.3 ± 10.4	2.33^*^	0.023^*^
Median EDSS (range)	–	2.0 (0–6)	3.5 (0–6.5)	3.35^§^	<0.001^§^

CIS, clinically isolated syndrome; MS, multiple sclerosis; MMSE = Mini-Mental State Examination; PASAT = Paced Auditory Serial Attention Test; EDSS = Expanded Disability Status Scale; TWMLL = Total White Matter Lesion Load. Data are mean ± standard deviation, except for EDSS presented in median (range). ^#^P value and F value were obtained by one-way analysis of variance. *P value and T value were obtained by two-sample t-test. ^╫^P value and χ2 value were obtained by χ2 test. ^§^P value and Z value were obtained by Wilcoxon rank sum test.
