# Supplementary material for: Disrupted topological organization of structural and functional brain connectomes in clinically isolated syndrome and multiple sclerosis
Source: Sci Rep. 2016 Jul 12;6:29383. doi: 10.1038/srep29383 (PMC4941534; doi:10.1038/srep29383)
Supplement: Supplementary Information [file srep29383-s1.doc]

**Supplemental Text**

**Text S1. Definitions of network metrics**

*Network strength.* For a network (graph) G with N nodes and K edges, we calculated the strength of G as:

$$S_{p}(G)=\frac{1}{N}\sum_{i\in G} S(i)$$

where S(i) is the sum of the edge weights w_ij_ linking to node i. The strength of a network is the average of the strength across all of the nodes in the network.

*Small-world properties.* Small-world network parameters (clustering coefficient, C_p_ and shortest path length, L_p_) were originally proposed by Watts and Strogatz ^1^. In this study, we investigated the small-world properties of the weighted brain networks.

The clustering coefficient of a node i, C(i), which was defined as the likelihood of whether the neighborhoods were connected with each other or not, was computed as follows ^2^:

$$C(i)=\frac{2}{k_{i}(k_{i}-1)}\sum_{j,k} (\bar{w}_{ij}\bar{w}_{jk}\bar{w}_{ki})^{1/3}$$

where k_i_ is the degree of node i and $\bar{w}$ is the weight of edge, which is scaled by the largest weight of the network. The clustering coefficient is zero if the nodes are isolated or have just one connection, i.e., k_i_ = 0 or k_i_ = 1. The clustering coefficient, C_p_, of a network is the average of the clustering coefficient over all nodes and indicates the extent of the local interconnectivity or cliquishness in a network ^1^.

The path length between any pair of nodes (e.g., node i and node j) is defined as the sum of the edge lengths along this path. For weighted networks, the length of each edge was assigned by computing the reciprocal of the edge weight, 1/w_ij_. The shortest path length, L_ij_, is defined as the length of the path for node i and node j with the shortest length. The shortest path length of a network was computed as follows:

$$L_{p}(G)=\frac{1}{N(N-1)}\sum_{i\neq j\in G} L_{ij}$$

where N is the number of nodes in the network. The L_p_ of a network quantifies the ability for information to propagate in parallel.

To examine the small-world properties, the clustering coefficient, C_p_, and the shortest path length, L_p_, of the brain networks were compared with those of random networks. In this study, we generated 100 matched random networks, which had the same number of nodes, edges, and degree distribution as the real networks ^3^. Of note, we retained the weight of each edge during the randomization procedure such that the weight distribution of the network was preserved. Furthermore, we computed the normalized L_p_, $\lambda=L_{p}^{real}/L_{p}^{rand}$, and the normalized C_p_, $\gamma=C_{p}^{real}/C_{p}^{rand}$, where $L_{p}^{rand}$ and $C_{p}^{rand}$ are the mean L_p_ and the mean C_p_ of 100 matched random networks, respectively. Importantly, two parameters correct the differences in the edge number and degree distribution of the networks across individuals. A real network would be considered small-world if $\gamma>1$ and $\lambda\approx1$ ^1^. Thus, a small-world network not only has a higher local interconnectivity, but it also has an approximately equivalent shortest path length compared with random networks. These two measurements can be summarized into a simple quantitative metric, small-worldness,$\sigma=\gamma/\lambda$, which is typically greater than 1 for small-world networks ^4^.

*Network efficiency*. The global efficiency of G measures the global efficiency of the parallel information transfer in the network ^5^, which can be computed as:

$$E_{glob}(G)=\frac{1}{N(N-1)}\sum_{i\neq j\in G} \frac{1}{L_{ij}}$$

where L_ij_ is the shortest path length between node i and node j in G.

The local efficiency of G reveals how much the network is fault tolerant and shows how efficient the communication is among the first neighbors of the node i when it is removed. The local efficiency of a graph is defined as:

$$E_{loc}(G)=\frac{1}{N}\sum_{i\in G} E_{glob}(G_{i})$$

where G_i_ denotes the subgraph composed of the nearest neighbors of node i.

*Regional nodal characteristics.* To determine the nodal (regional) characteristics of the brain networks, we computed the nodal efficiency, E_nodal_(i), which is defined as ^6^:

$$E_{nodal}(i)=\frac{1}{N-1}\sum_{i\neq j\in G} \frac{1}{L_{ij}}$$

where L_ij_ is the shortest path length between node i and node j in G. E_nodal_(i) measures the average shortest path length between a given node i and all of the other nodes in the network.

**References**

1. Watts, D.J. & Strogatz, S.H. Collective dynamics of 'small-world' networks. *Nature* **393**, 440-442 (1998).

2. Onnela, J.P., Saramaki, J., Kertesz, J. & Kaski, K. Intensity and coherence of motifs in weighted complex networks. *Phys Rev E Stat Nonlin Soft Matter Phys* **71**, 065103 (2005).

3. Maslov, S. & Sneppen, K. Specificity and stability in topology of protein networks. *Science* **296**, 910-913 (2002).

4. Humphries, M.D. & Gurney, K. Network 'small-world-ness': a quantitative method for determining canonical network equivalence. *PLoS ONE* **3**, e0002051 (2008).

5. Latora, V. & Marchiori, M. Efficient behavior of small-world networks. *Physical review letters* **87**, 198701 (2001).

6. Achard, S. & Bullmore, E. Efficiency and cost of economical brain functional networks. *PLoS computational biology* **3**, e17 (2007).

### Table S1. Cortical and subcortical regions of interest defined in the study

| **Index** | **Regions** | **Abbr.** | **Index** | **Regions** | **Abbr.** |
| --- | --- | --- | --- | --- | --- |
| (1,2) | Precental gyrus | PreCG | (47,48) | Lingual gyrus | LING |
| (3,4) | Superior frontal gyrus, dorsolateral | SFGdor | (49,50) | Superior occipital gyrus | SOG |
| (5,6) | Superior frontal gyrus, orbital part | ORBsup | (51,52) | Middle occipital gyrus | MOG |
| (7,8) | Middle frontal gyrus | MFG | (53,54) | Inferior occipital gyrus | IOG |
| (9, 10) | Middle frontal gyrus, orbital part | ORBmid | (55,56) | Fusiform gyrus | FFG |
| (11,12) | Inferior frontal gyrus, opercular part | IFGoperc | (57,58) | Postcentral gyrus | PoCG |
| (13,14) | Inferior frontal gyrus, triangular part | IFGtriang | (59,60) | Superior parietal gyrus | SPG |
| (15,16) | Inferior frontal gyrus, orbital part | ORBinf | (61,62) | Inferior parietal, but supramarginal and angular gyri | IPL |
| (17,18) | Rolandic operculum | ROL | (63,64) | Supramarginal gyrus | SMG |
| (19,20) | Supplementary motor area | SMA | (65,66) | Angular gyrus | ANG |
| (21,22) | Olfactory cortex | OLF | (67,68) | Precuneus | PCUN |
| (23,24) | Superior frontal gyrus, medial | SFGmed | (69,70) | Paracentral lobule | PCL |
| (25,26) | Superior frontal gyrus, medial orbital | ORBsupmed | (71,72) | Caudate nucleus | CAU |
| (27,28) | Gyrus rectus | REC | (73,74) | Lenticular nucleus, putamen | PUT |
| (29,30) | Insula | INS | (75,76) | Lenticular nucleus, pallidum | PAL |
| (31,32) | Anterior cingulate and paracingulate gyri | ACG | (77,78) | Thalamus | THA |
| (33,34) | Median cingulate and paracingulate gyri | DCG | (79,80) | Heschl gyrus | HES |
| (35,36) | Posterior cingulate gyrus | PCG | (81,82) | Superior temporal gyrus | STG |
| (37,38) | Hippocampus | HIP | (83,84) | Temporal pole: superior temporal gyrus | TPOsup |
| (39,40) | Parahippocampal gyrus | PHG | (85,86) | Middle temporal gyrus | MTG |
| (41,42) | Amygdala | AMYG | (87,88) | Temporal pole: middle temporal gyrus | TPOmid |
| (43,44) | Calcarine fissure and surrounding cortex | CAL | (89,90) | Inferior temporal gyrus | ITG |
| (45,46) | Cuneus | CUN |  |  |  |

Note: The regions are listed in terms of a prior template of an AAL-atlas (Tzourio-Mazoyer et al., 2002).
